# Navigating the sustainability landscape: a systematic review of sustainability approaches in healthcare

**DOI:** 10.1186/s13012-017-0707-4

**Published:** 2018-02-09

**Authors:** L. Lennox, L. Maher, J. Reed

**Affiliations:** 1 NIHR CLAHRC North West London, 369 Fulham Road, London, SW10 9NH United Kingdom; 2 Department of Primary Care and Public Health, Imperial College London, 369 Fulham Road, London, United Kingdom; 3 Ko Awatea I Health System Innovation and Improvement, Middlemore Hospital, 100 Hospital Road, Otahuhu, New Zealand

**Keywords:** Sustainability, Method, Tool, Model, Framework, Assessment, Quality improvement

## Abstract

**Background:**

Improvement initiatives offer a valuable mechanism for delivering and testing innovations in healthcare settings. Many of these initiatives deliver meaningful and necessary changes to patient care and outcomes. However, many improvement initiatives fail to sustain to a point where their full benefits can be realised. This has led many researchers and healthcare practitioners to develop frameworks, models and tools to support and monitor sustainability. This work aimed to identify what approaches are available to assess and influence sustainability in healthcare and to describe the different perspectives, applications and constructs within these approaches to guide their future use.

**Methods:**

A systematic review was carried out following PRISMA guidelines to identify publications that reported approaches to support or influence sustainability in healthcare. Eligibility criteria were defined through an iterative process in which two reviewers independently assessed 20% of articles to test the objectivity of the selection criteria. Data were extracted from the identified articles, and a template analysis was undertaken to identify and assess the sustainability constructs within each reported approach.

**Results:**

The search strategy identified 1748 publications with 227 articles retrieved in full text for full documentary analysis. In total, 62 publications identifying a sustainability approach were included in this review (32 frameworks, 16 models, 8 tools, 4 strategies, 1 checklist and 1 process). Constructs across approaches were compared and 40 individual constructs for sustainability were found. Comparison across approaches demonstrated consistent constructs were seen regardless of proposed interventions, setting or level of application with 6 constructs included in 75% of the approaches. Although similarities were found, no approaches contained the same combination of the constructs nor did any single approach capture all identified constructs. From these results, a consolidated framework for sustainability constructs in healthcare was developed.

**Conclusions:**

Choosing a sustainability method can pose a challenge because of the diverse approaches reported in the literature. This review provides a valuable resource to researchers, healthcare professionals and improvement practitioners by providing a summary of available sustainability approaches and their characteristics.

**Trial registration:**

This review was registered on the PROSPERO database: CRD42016040081 in June 2016.

**Electronic supplementary material:**

The online version of this article (10.1186/s13012-017-0707-4) contains supplementary material, which is available to authorized users.

## Background

Internationally, there is a need to continually improve health and care services. To support this, many healthcare organisations are engaged in a wide range of improvement initiatives. Despite the significant investment of staff time and other resources, many promising initiatives fail to sustain and do not produce long term benefits [1–6]. Sustaining worthwhile changes poses a challenge to those undertaking an improvement initiative. A systematic review of 125 studies of improvements made in healthcare found that the projects do not maintain all aspects originally implemented with fewer than half continuing interventions at high levels of fidelity [1]. Similar results were found in a review on the continuation of programme activities where only 60% of sites reported sustaining at least one programme component [5].

Initiatives that fail to sustain are extremely wasteful of human and monetary investments [7, 8]. Large variation in the practices and care can be seen across similar services when initiatives which initially demonstrate improved patient outcomes fail to maintain their gains [7, 8]. This has also been shown to be detrimental to improvement efforts in general as staff, patient and public opinion of improvement initiatives declines and enthusiasm for engaging in future programmes is lost [9, 10]. In the current climate of rising demands, shifting priorities and competition for resources, there is a need to understand how sustainability of implemented initiatives can be influenced as health planners and other stakeholders want to ensure the long-term impact of their investments [1, 11]. Despite recognition of this challenge and considerable research conducted in this area, relatively little is known about how to translate this evidence into action to support the long-term impact of improvement efforts [12].

Further complicating this research area is the lack of consensus on how to define sustainability. This has led to contradictory recommendations for influencing sustainability and debate on what qualifies as a sustained improvement [10, 13]. Sustainability is often viewed as an ‘outcome’ where health benefits, activities or workforce capacity are maintained [8]. Some have cautioned against this linear perspective on sustainability as it ‘does not take account of the recursive or reflexive character of sustainability and learning or of the continuous adjustments that shape the sustainability process’ [13]. More recently, the ability to adapt and continuously improve has also been recognised as a potential definition of sustainability [14]. This concept of sustainability as a ‘process’ rather than an ‘outcome’, incorporates concepts of adaptation, learning and continuous development [15]. This lens allows sustainability to be viewed as a change process that can be influenced by individuals throughout initiatives by continuing to develop and adapt in response to the needs of the system [15–17]. For the purposes of this work, ‘sustainability’ will refer to the general continuation and maintenance of a desirable feature of an initiative and its associated outcomes as well as the process taken to adapt and develop in response to emerging needs of the system. This definition as well as any additional domains found will be explored in the review.

With no clear consensus on how to define or influence sustainability, many researchers and healthcare practitioners have developed frameworks, models and tools to support and monitor sustainability in healthcare settings [12, 18]. With little overarching direction for this area of research, new definitions, factors and methods for assessing sustainability have been produced by individual studies [18]. Some work has been undertaken to review frameworks for sustainability in specific settings and programmes, but little has been done to comprehensively review available approaches for sustainability across healthcare settings [15, 18, 19]. It is recognised that diverse healthcare settings ‘use similar processes to achieve adoption, implementation, and sustainability’ which indicates general learning and lessons can be gathered from across settings to inform sustainability research [18]. This provides an opportunity to draw from the current literature to investigate available sustainability approaches and develop a sustainability knowledge base that is useful beyond specific settings or interventions [18, 20]. This paper offers a review of sustainability approaches to support healthcare teams and researchers to understand the different perspectives, applications, and constructs within approaches to guide their use in healthcare improvement initiatives.

This review addressed the following research questions:

1. What approaches have been proposed to influence or assess sustainability in healthcare?
2. Where have they come from and how have they been developed?
3. What are their key characteristics?
4. What sustainability constructs are examined in each approach?

## Methods

### Search and information sources

A systematic review was undertaken guided by the Preferred Reporting Items for Systematic Reviews and Meta-Analysis (PRISMA) reporting standards [21]. The selection of databases, search terms and search strategy was supported by a medical librarian to ensure an overall quality and coverage of the systematic review. The search was carried out on Embase, HMIC Health Management Information Consortium, and Ovid MEDLINE in January 2017, and a follow-up search was conducted prior to submission in September 2017. Key words included a combination of sustainability terms (sustain*, institutionali#ation, routini#ation, maintenance, integration, normali#ation, embed*) and method terms (model, framework, tool, plan, checklist, scale, strategy, theory, conceptuali#ation) along with health or healthcare. A snowballing approach was also taken; references from included papers were analysed and retrieved if deemed relevant.

### Data collection process and study selection

We sought approaches (for the purposes of this work, the term approaches refers to published models, checklists, tools, processes, strategies, conceptualisations and frameworks) that aim to influence and/or assess sustainability within healthcare settings. The level or type of influence was not specified but could include assessment, planning, evaluation, monitoring, prediction or testing. Papers published in peer-reviewed journals introducing a tangible and clear approach for sustainability were included. Papers published in languages other than English were excluded. Approaches used within a larger system process or staged process (for example an implementation model including sustainability as the final stage) were excluded. Commentary, posters, protocols, conference proceedings, editorials and perspectives were excluded. Papers only defining or constructing concepts of sustainability were excluded. Two authors independently screened the first 20% of the full-text articles for inclusion. Any differences in selected articles were discussed, and inclusion and exclusion criteria were refined to reflect these discussions. One author (LL) then screened the remaining papers for inclusion.

### Quality assessment and data extraction strategy

A quality assessment and data extraction form was developed for identified articles. Existing quality assessments were explored, but it has been noted that available quality assessment approaches often fail to consider the rationale and context of studies [22, 23]. Their use to determine the inclusion of qualitative studies is often not recommended as many existing tools do not capture the multiple meanings of “good quality” and “rightness”; therefore, studies should often not be excluded based on this quality assessment [22, 24]. The available assessments were not sensitive to the aim of our study which was exploratory in nature. We sought to provide an overview of available approaches for sustainability and designed our data extraction form to identify and describe the included articles. The aim of the data extraction was to report descriptions and study information not to ascertain validity of the approaches or their constructs. To ensure the studies met the baseline quality expected, each article was assessed with the structured data extraction form. Data extraction included strategy name, purpose of use, healthcare setting, level of healthcare use, description of use, sustainability constructs, scoring mechanism, target user, definition of sustainability, theoretical underpinning, sustainability perspective and method development details. One author (LL) extracted the data from the articles. This information was then independently checked against the full-text articles by the second author (LM). Any missing data or discrepancies were discussed between authors and were resolved by consensus. Agreement was reached for accuracy of all studies.

### Data synthesis and presentation

To examine the sustainability constructs within each method, articles were uploaded to Nvivo 10 software for analysis. Template analysis was conducted using predefined codes to guide the analysis process [25]. Constructs within one method (Shediac-Rizkallah and Bone’s conceptual sustainability framework) served as the baseline template for coding sustainability constructs [8]. This technique allowed each approach’s constructs to be compared and contrasted and additional constructs to be identified. The preliminary coding structure was iteratively developed with new constructs integrated and refined as further sustainability approaches were added to the dataset. One author conducted the initial coding with input from other authors on coding structure and construct labels. To assess coding clarity and reliability, a second coder independently coded 25% of the articles and an inter-rater reliability score (kappa coefficient) was calculated. Discrepancies between coders were used to refine codes and revise the definitions and inclusion criteria for each of the constructs. Results have been summarised using ratios and narrative summaries.

### Risk of bias in individual studies and across studies

This review aimed to explore the creation and introduction of sustainability approaches; therefore, results other than the description of the sustainability method in individual studies were not analysed. As this review focused on published sustainability approaches, publication bias may have affected the results of this study. Approaches available in the grey literature were identified but not included in this review.

### Registration

This systematic review was registered on the PROSPERO database under the registration number: CRD 42016040081 in June 2016 [26].

## Results

**Fig. 1:**
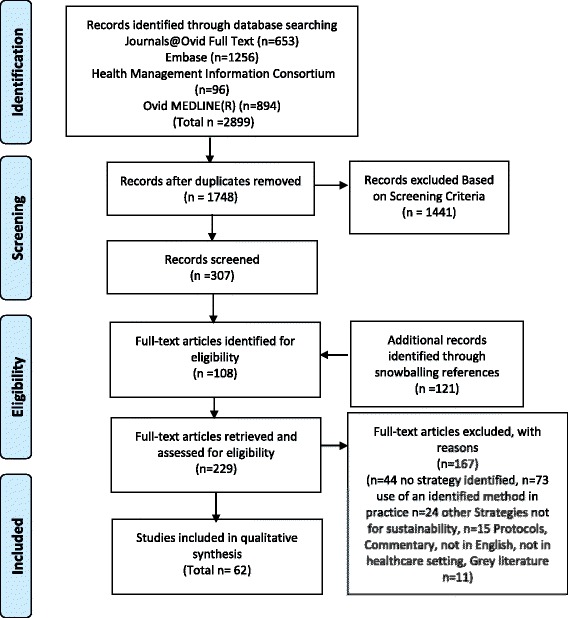
PRISMA diagram. Description of search strategy and article retrieval

The search strategy resulted in 2889 publications from the databases. Snowballing and electronic citation tracking identified 121 further papers for potential inclusion. Titles and abstracts were examined, and 229 articles were retrieved in full text for full documentary analysis. In total, 62 papers which identified sustainability approaches were identified for inclusion in this review Fig. 1.

### Sustainability approaches

**Fig. 2:**
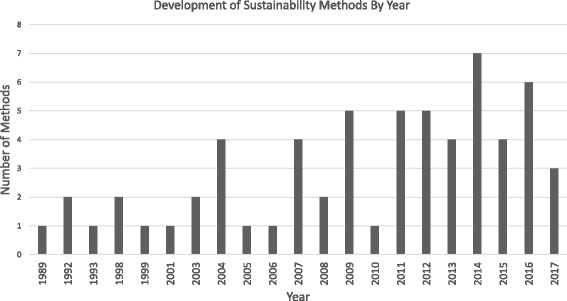
Development of sustainability approaches by year. Graph displays sustainability approach development by year

The 62 papers identifying sustainability approaches are outlined in Table 1. Full data extraction details for each approach are available in Additional file 1. Sustainability approaches have been consistently developed and adapted since the late 1980s with an average of two created every year Fig. 2.

**Table 1:** Papers included in review

Author	Year	Name	Purpose
1. Alexander, J.A. et al. [62]	2003	The model for community health partnership sustainability	To provide practical guidelines for partnership sustainability
2. Amaya, A. et al. [65]	2014	Conceptual framework for sustainability	To identify themes and relationships emerging from data to identify recommendations to inform decision-makers on priorities
3. Ament, S. et al. [80]	2014	Strategies to sustain improvements in hospital practice	To suggest post-implementation strategies which are valuable in sustaining implementation successes
4. Atun, R. et al. [53]	2010	A conceptual framework for analysing integration of health interventions into health systems	To analyse and map the nature and extent of integration in different settings, along with the factors that influence the integration process
5. Azeredo, B.T. et al. [45]	2017	Framework for investigating the sustainability of ARV provision	To structure data collection and analysis
6. Blackford, J. and Street, A [69]	2012	The Advance Care Planning-Service Evaluation Tool (ACP-SET)	To assist community-based palliative care services to establish a sustainable system-wide model relevant to their local context
7. Blanchet, K. and Girois, S [57]	2013	The Sustainability Analysis Process (SAP)	To conceptualise and measure sustainability of health systems in low-income countries and fragile states
8. Bray, P. et al. [81]	2009	Sustainability Pyramid Model	To propose a series of practice characteristics that constitute critical elements for QI sustainability activities
9. Brinkerhoff, D. and Goldsmith, A. [20]	1992	The analytical framework for Institutional sustainability	To analyse the generic conditions for sustaining institutions in general and provide suggested strategies
10. Chambers, D. et al. [11]	2013	The Dynamic Sustainability Framework	To maximise the fit between interventions, practice settings and the broader ecological system over time
11. Dauphinee, W. and Reznick, R [63]	2011	Framework for guiding change and managing and monitoring a successful multicentered network.	To identify success factors that can facilitate the adoption of a national simulation network
12. Dominick, G.M. et al. [82]	2016	ENRICH Sustainability Survey	To identify residential children’s homes (RCHs) that sustained PA-promoting environments.
13. Dorsey, S. et al. [46]	2014	NINR Logic Model for Center Sustainability	To provide guidance for those who wish to develop and sustain a centre or plan for sustainability
14. Edwards, J. C. et al. [42]	2007	Catholic Healthcare partners HF-GAP Sustainability Assessment (AHRQ)	To trigger planning for sustainability early in a project’s design
15. Feldstein, A.C. and Glasgow, R.E [83]	2008	Practical, Robust Implementation and Sustainability Model (PRISM)	To enhance implementation and sustainability and to help conceptualise, implement and evaluate health care improvement programmes
16. Finch, T.L. et al. [84]	2012	Technology Adoption Readiness Scale (TARS)	To contribute to the successful normalisation of e-health, either as a ‘diagnostic’ tool or for evaluation purposes
17. Fleiszer et al. [58]	2015	Framework for the sustainability of healthcare innovations	To guide data collection and content analysis
18. Ford, J.H. et al. [47]	2015	Strategies to Sustain Use of A-CHESS	To suggest strategies to be used to sustain the use a mobile app
19. Fox, A. et al. [79]	2015	The sustainability of innovation theoretical framework	To guide research, determine variables, influence data analysis
20. Goodman et al. [85]	1993	Level of Institutionalisation (LoIn) Scale	To measure the extent of programme integration into an organisation
21. Goodman, R. and Steckler, A [86]	1989	Model for Program Institutionalisation	To demonstrate how health promotion programmes may become institutionalised to guide programme design and evaluation
22. Gruen, R.L. et al. [7]	2008	Model of health-programme sustainability	To provide a model of health-programme sustainability based on context and resource availability
23. Hanson, D. et al. [43]	2005	A systematic ecological framework to design sustainable interventions	To design sustainable, community-based, safety promotion interventions
24. Hodge L.M. and Turn, K [54]	2016	A Conceptual Framework of Supporting Factors	To guide and evaluate capacity building in EBP implementation and sustainment in low-resource community settings
25. Isabalija, S.R. et al. [87]	2013	Framework for e-medicine sustainability	To facilitate the development, implementation, and sustainability of e-medicine by providing professionals with information on which to build their sustainability efforts
26. Iwelunmor, J. et al. [68]	2016	A conceptual framework	To bring attention to sustainability as a core component embedded within the overall life cycle of an intervention that evolves through time
27. Johnson et al. [19]	2004	A Sustainability Planning Model	To address two sets of sustainability factors known to be associated with success in sustaining an innovation
28. Knight, T. et al. [59]	2001	A framework for evaluating the sustainability of collaborative working	To provide formative evaluation of future collaborative initiatives and analysis of collaborative working
29. Leffers, J. and Mitchell, E [88]	2011	Conceptual Framework for Partnership and Sustainability in Global Health Nursing.	To offer guidance and a framework for partnership and sustainability for nurses who participate in global efforts
30. Lennox et al. [56]	2017	The Long Term Success Tool (LTST)	To support those implementing improvements reflect on 12 key factors to identify risks and prompt actions to increase chances of sustainability over time
31. Luke, D.A. [36]	2014	Program Sustainability Assessment Tool (PSAT)	To assess and plan for sustainability risks and develop an action plans
32. Maher, L. et al. [61]	2010	NHS III Sustainability Model	To predict the likelihood of sustainability and guide teams to things they could do to increase the chances that changes will be sustained
33. Mancini, J.A. and Marek, L.I [37]	2004	Model of community-based program sustainability/Program Sustainability Index (PSI)	To evaluate community-based programme sustainability
34. May, C. and Finch, T [89]	2009	Normalisation Process Theory	To explore the social organisation of the work (implementation), of making practices routine elements of everyday life (embedding), and of sustaining embedded practices in their social contexts (integration)
35. May, C. et al. [51]	2006	Normalisation Process Model	To assist in explaining the processes by which complex interventions become routinely embedded in health care practice
36. Melnyk, B. and Fineout-Overholt, E [90]	2011	The ARCC (Advancing Research and Clinical practice through close Collaboration) model	To provide health care systems with a conceptual framework to guide system-wide implementation and sustainability of EBP for the purpose of improving quality of care and patient outcomes
37. Nelson, D.E. et at [39]	2007	The five basic elements of program sustainability	To suggest five basic elements of programme sustainability for tobacco control programmes, to understand the factors associated with success
38. Nystrom, M.E. et al. [91]	2014	Strategies to facilitate implementation and sustainability of large system transformations	To provide an approach to implement and sustain a large national change programme
39. Okeibunor, J. et al. [60]	2012	A model for evaluating the sustainability of community-directed treatment	To provide critical indicators of project performance to evaluate sustainability
40. Olsen, I. T [92]	1998	Sustainability of health care: A framework for analysis	To study the sustainability of health services in developing countries
41. Parand, A [38]	2012	Strategies to sustain Safer Patient Initiative (SPI)	To recommend strategies to facilitate the sustainability of a quality and safety improvement collaborative
42. Persaud, D [52]	2014	The ELIAS (Enhancing Learning, Innovation, Adaptation, and Sustainability) Performance Management Framework	To improve the sustainability of healthcare organisations
43. Rasschaert, F. et al. [93]	2014	Conceptual framework on sustainability of community-based programmes	To explore the data retrieved and to identify factors influencing the sustainability
44. Racine, D.P [66]	2006	Model of sustaining innovations in their effectiveness	To suggest a comprehensive conceptual framework of programmatic, organisational and environmental factors that may shape the circumstances for sustaining and replicating effectiveness
45. Roy, M. et al. [48]	2016	Framework for Sustained Retention	To understand sustained retention, highlight barriers specific to sustained retention and review interventions addressing long-term, sustained retention
46. Rudd, R. E. et al. [94]	1999	A five-stage model for sustaining a community campaign	The five-stage model offers a mechanism for expanding the life of a campaign
47. Sarriot, E.G. et al. [31]	2004	Child Survival Sustainability Assessment (CSSA) framework and process	To provide a process for a participatory sustainability assessment with communities and local partners
48. Sarriot, E.G. et al. [28]	2008	The Sustainability Framework	To organise thinking about sustainability as well as inform planning, management, and evaluation of activities in order to improve and maintain health outcomes at a population level
49. Saunders, R.P [64]	2012	LEAP Sustainability Assessment	To assess sustainability of the Lifestyle Education for Activity Program (LEAP)
50. Savaya, R [49]	2009	Projected Likelihood of Project’s Continuation	To examine projected sustainability and its predictors along a continuum of forms
51. Schalock, R. et al. [30]	2016	Sustainability model	To consider what factors drive the organisation’s ability to both adapt successfully to change
52. Scheirer, M. and Dearing, J.W [18]	2011	A Generic Conceptual Framework for Sustainability	To guide the sustainability research agenda
53. Schell, S.F. et al. [44]	2013	Capacity for sustainability framework	To provide a framework on sustainability capacity, identifying organisational and contextual characteristics necessary for successfully sustaining programmes over time
54. Shediac-Rizkallah, M.C. & Bone, L.R [8]	1998	Conceptual framework for planning for sustainability of community based health programs	To conceptualise and measure sustainability and provide guidelines to facilitate sustainability in community programmes
55. Shigayeva, A. and Coker, R [15]	2015	Conceptual framework to support analyses of sustainability	To support analyses of sustainability of communicable disease programmes
56. Sivaram, S. and Celentano, D.D [27]	2003	Conceptual framework to develop a strategy that will facilitate sustainability	To develop a strategy that will facilitate sustainability of outreach worker efforts in AIDS prevention
57. Slaghuis, S.S. et al. [32]	2011	A framework and a measurement instrument for sustainability of work practices in long-term care	To analyse sustainability of actual changed work practices and evaluate improvement projects
58. Song, B. et al. [50]	2016	The framework for sustainability evaluation of Community based LTC programmes	To evaluating community-based LTC programmes from the sustainability perspective
59. Sridharan, S. et al. [29]	2007	Analysis of strategic plans to assess planning for sustainability of comprehensive community initiatives	To assess planning for sustainability
60. Stefanini, A. and Ruck, N [41]	1992	Conceptual framework to monitor the performance of externally-assisted health projects	To monitor a project’s efforts towards sustainability
61. Story et al. [67]	2017	Conceptual framework for institutionalization of community-focused maternal, newborn & child health strategies	To encourage collaboration and contribute to programme planning and policy making for the institutionalisation of community-focused health strategies
62. Tuyet Hanh, T.T. et al. [40]	2009	Framework for Evaluating the Sustainability of Community-based Dengue Control Projects	To provide a framework and tool for assessing sustainability

### Theoretical perspectives, definitions and development details

#### Theoretical perspectives

Exploring the theoretical underpinnings of the approaches revealed diverse theoretical grounding. Although 37% (23/62) did not have an explicit link to theory, 15 different theories were identified within the other approaches. While numerous theories were found, 4 theories were common across multiple sustainability approaches covering 45% of papers: *diffusion of innovations theory*, *complexity theory*, *ecological theory* and *open systems theory*. Theoretical perspectives guided how sustainability was defined within approaches and how it was viewed within healthcare systems. A brief description of the most common perspectives and their links to the sustainability approaches are outlined in Table 2.

**Table 2:** Theoretical perspectives

	Diffusion of innovations [70, 95]	Complexity theory/complex systems theory [71]	Ecological theory [72, 96]	General systems theory or open systems theory [73]
No. of approaches drawing on theory	10	9	5	4
Sustainability process	Sustainability is viewed as the final stage of initiative life cycle [18, 86]	Sustainability is a nonlinear process where change, adaptation and uncertainty are expected [15, 31, 53, 68]	Views sustainability as an ongoing and dynamic process that occurs throughout implementation [11, 72]	Sustainability is a process where things can return to the norm (‘homeostasis’) or adapt to the environment to survive. [85, 92]
Theory application in approaches	This perspective explores how programme benefits and burden will support or be a barrier to sustainability [54, 66]. Within approaches using this perspective, the role of adopters of the initiatives were seen as key to success, specifically to achieve wider reach during initiatives and maintain activities after the initiatives come to an end [27].	This perspective highlights how the interactions that occur between an initiative, the setting, the broader organisation and the sociocultural context impact sustained change. Initiatives were viewed as components being introduced to complex adaptive systems that change and adapt in response to interactions with the environments, individuals and wider context [53].	This perspective focuses on behaviour and how it is influenced by and influences individuals and environments [72] Approaches adopting this perspective focused on the need to find the right fit between initiatives, contexts and expectations to inform the ongoing adaptation of initiatives to achieve sustainability [11]	This perspective views an organisation as an organism open to the influence of its environment with the need to adapt to survive in order to achieve lasting change [73] Approaches using this perspective explored perceived benefits and burden of an initiatives, availability of support for initiatives and leadership within organisations [54]

#### Definitions of sustainability

Definitions for sustainability were explicitly stated in 76% (47/62) of approaches and implicitly deduced from the remaining 24%. Multiple definitions were found across approaches, but 5 distinct definitions for sustainability were identified:

1. *Continued programme activities* (included in 86% (53/62) of the approaches)

- e.g. ‘The ability of activities to continue appropriate to the local context after withdrawal of external funding’ [27].

2. *Continued health benefits* (included in 44% (27/62))

- e.g. ‘Sustainability is the ability to sustain population health outcomes.’ [28]

3. *Capacity built* (included in 19% (12/62))

- e.g. ‘our conceptualization of sustainability was on the inter-organizational relationships that might serve as a basis of the collaborative problem-solving capacity’ [29].

4. *Further development* (*adaptation*) (included in 16% (10/62))

- e.g. ‘Adapting successfully to change and providing a range of valued service delivery opportunities and practices in an effective and efficient manner’ [30].

5. *Recovering costs* (included in 3% (2/62))

- e.g. ‘It is the ability of an organization to produce outputs of sufficient value so that it acquires enough inputs to continue production at a steady or growing rate’ [20].

#### Sustainability approach development

**Fig. 3:**
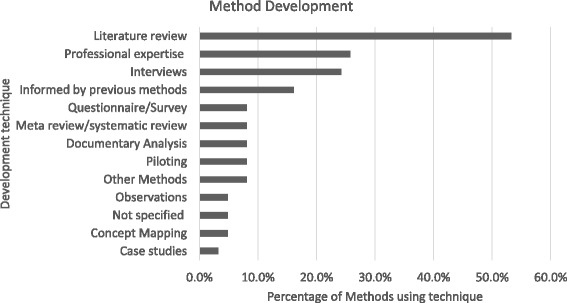
Sustainability approach development techniques. Development techniques used to create sustainability approaches

The sustainability approaches were developed through several techniques often using a mixed-method approach (e.g. literature review and interviews) (Fig. 3). Sixty-one percent (38/62) of the development processes included a literature review or systematic review. This was followed by 26% (16/62) using ‘professional expertise’ such as an advisory panel and 24% (15/62) using interviews.

### Sustainability method characteristics

#### Type

The sustainability approaches come in a variety of forms: frameworks/conceptual frameworks (32), models (16), tools (8), guidance strategies (4), checklists (1) and processes (1). The highest proportion identified themselves as frameworks. Our exploration indicates there is very little consensus between approaches on what constitutes a ‘framework’, ‘model’ or ‘tool’.

#### Aim

The highest proportion of approaches, 39% (24/62), aimed to *evaluate* sustainability, followed by 23% (14/62) of the approaches which aimed to support *planning* for sustainability. The remaining approaches aimed to provide *guidance* and *strategies* to influence sustainability or a combination of *evaluation*, *planning and guidance.*

#### When to assess

Two distinct perspectives on when approaches should be used emerged from this review. The highest proportion, 66% (41/62) of approaches, viewed the sustainability as a prospective process to be explored throughout implementation. Nine approaches viewed sustainability as a linear process with sustainability being studied retrospectively after implementation has been ‘completed’. The remaining 12 approaches specified they could be used both prospectively and retrospectively, during implementation or following implementation.

#### Level of use

The majority, 82% (51/62), of approaches have been designed to examine or influence sustainability at a specific *intervention or programme level* (e.g. a single improvement project) [31]. Eleven approaches aimed to examine sustainability at an *organisational or systems level (e.g. a long-term care organisation)* [32].

#### Settings

Thirty-seven percent (23/62) of the approaches were designed for use in general healthcare settings and did not specify a specific healthcare setting for use. Public health settings were specified in 31% (19/62) of the approaches, followed by community healthcare in 26% of the approaches (16/62). A smaller number of approaches were designed for use in acute, 3% (2/62), and e-health settings, 3% (2/62).

#### Suggested users

**Fig. 4:**
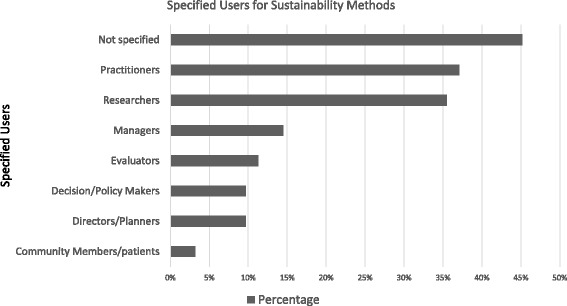
Suggested users for approaches

Suggested users were specified in 55% (34/62) of the approaches (Fig. 4). The majority of these approaches have been designed for use by multiple groups of professionals or practitioners (e.g. researchers as well as nurses).

### Sustainability constructs

Constructs across approaches were compared and contrasted, and 40 individual items for sustainability were found. The number of constructs examined in each method ranged from 8 to 31 with an average of 17 constructs per method. Additional file 2 provides a description of inclusion, a definition and an example for each of the 40 constructs. To assess coding clarity and reliability, an inter-rater reliability score (kappa coefficient) was calculated between two coders using the NVivoPro coding comparison function [33]. The test showed a high level of agreement between scorers with an inter-rater reliability score of 0.94 [34, 35].

A consolidated framework for sustainability constructs in healthcare is presented in Table 3 and summarises the frequency of sustainability constructs across the approaches. The constructs have been organised under the following six emergent themes: *the initiative design and delivery*, *negotiating initiative processes*, *the people involved*, *resources*, *the external environment and the organisational setting*. Comparison across approaches demonstrated that no two approaches contained the same combination of the constructs nor did any single approach capture all 40 constructs. Although variation was seen, results show that there are consistent constructs across approaches regardless of proposed interventions, settings or application types. Six constructs were included in over 75% of the approaches: ‘General resources’ (90%), ‘Demonstrating effectiveness’ (89%), ‘Monitoring progress over time’ (84%), ‘Stakeholder participation’ (79%), ‘Integration with existing programs and policies’ (79%) and ‘Training and capacity building’ (76%).

**Table 3:** Consolidated framework for sustainability constructs in healthcare

The initiative design and delivery	Negotiating initiative processes	The people involved	Resources	The organisational setting	The external environment
• Demonstrating effectiveness 89%	• Belief in the initiative 63%	• Stakeholder participation 79%	• General resources 90%	• Integration with existing programs and policies 79%	• Socioeconomic and political considerations 63%
• Monitoring progress over time 84%	• Accountability of roles and responsibilities 56%	• Leadership and champions 73%	• Funding 68%	• Intervention adaptation and receptivity 73%	• Awareness and raising the profile 45%
• Training and capacity building 76%	• Defining aims and shared vision 53%	• Relationships and collaboration and networks 65%	• Infrastructure 26%	• Organisational values and culture 71%	• Urgency 5%
• Evidence base for the initiative 52%	• Incentives 31%	• Community participation 56%	• Resource_Staff 26%	• Organisational readiness and capacity 56%	• Spread to other organisations 5%
• Expertise 23%	• Workload 27%	• Staff involvement 42%	• Resource_Time 6%	• Support available 40%	
• The problem 15%	• Complexity 24%	• Ownership 26%		• Opposition 5%	
• Project duration 8%	• Job requirements 19%	• Power 18%			
• Improvement methods 6%		• Patient involvement 16%			
• Project type 2%		• Satisfaction 11%			

#### Diversity in assessment

Although common constructs were found across approaches, each approach reported diverse means to investigating and defining individual constructs. As an exemplar, the top 3 most common constructs are presented in more detail to highlight how similar constructs are assessed across different approaches. Inclusion information and definitions for all constructs are available in Additional file 2.

- *Resources*. This construct included a complex combination of potential resources to consider. Four key resource types were found: *funding*, *infrastructure*, *staff* and *Time*. The majority of the approaches explicitly stated the need to assess resources but not all indicated the type of resource. Many approaches highlighted the importance of the ability of an initiative *to garner and maintain* resources [15, 27, 36–41] through *stable sources* [19, 36, 39, 42–45]. The ability of an initiative to *share resources* with partners and other organisations [41, 46], seek out *alternative and supplemental resources* [18, 47–49] and/or uncover *multiple funding sources* [8, 36, 49, 50] were also highlighted across some approaches as important to overall sustainability.
- *Demonstrating effectiveness* (assessing or measuring project outcomes and impact). A number of potential perspectives were taken to assess this construct. While some approaches chose to look at overall initiative evaluation or performance [32, 36, 44, 51, 52], others chose to specifically assess either the ability of the initiative to *function* as intended [15, 36, 39] or the ability of the initiative to *produce* intended benefits [7, 11, 31, 47, 53–60]. A selection of approaches took a wider perspective and looked at whether the initiative benefits were perceived by staff and other stakeholders as *valuable* [8, 30, 42, 51, 61-63].
- *Monitoring progress over-time* (the ability to monitor the initiative using standardised systems or mechanisms over-time) appeared in 84% of the approaches*.* Approaches to monitoring included diverse areas to assess including *having appropriate data to document progress* [64, 65], having *a management or monitoring system in place* [15, 53, 61, 66, 67], *and* having *regular reporting and feedback mechanisms* [46, 47, 52, 54, 68, 69].

### Top ten comparisons across approaches

#### Comparison across level of use

The top ten constructs for examining an *organisation or system’s* sustainability versus an *intervention or programme’s* sustainability are presented in Table 4. Regardless of level of use, 5 of the top 10 constructs are found across both types of approaches. Differences between these types of approaches demonstrate how the ‘level of use’ of an approach changes the potential constructs to be explored (shown in italics). In studying an *organisation or system’s sustainability*, there is a greater focus assessing the *readiness and capacity* for the initiatives and *involving stakeholders* and *community members*. Approaches assessing organisational sustainability were also much more likely to prioritise *defining overall aims* for the programme and garnering *belief in initiatives* from stakeholders. Approaches assessing an *intervention’s sustainability *emphasised the need to consider how an initiative becomes *integrated into current programmes and policies* specifically looking what *intervention adaption* may be needed. These approaches were also more likely to assess how *training and capacity building* were conducted to ensure staff were able to undertake the initiative tasks.

**Table 4:** Comparison across level of use (difference shown in italics)

Organisational focus (11 approaches)	Percent	Intervention focus (51 approaches)	Percent
1. Demonstrating effectiveness	100	1. Resources_General	90
2. Resources_General	91	2. Demonstrating effectiveness	86
3. Monitoring progress over time	91	3. Monitoring progress over time	82
4. *Organisational readiness and capacity*	82	4. *Integration with existing programs and policies*	82
5. *Belief in the initiative*	73	5. *Training and capacity building*	76
6. Organisational values and culture	73	6. Stakeholder participation	76
7. *Community participation*	73	7. *Intervention adaptation and receptivity*	75
8. Leadership and champions	73	8. Leadership and champions	73
9. *Stakeholder participation*	73	9. *Organisational values and culture*	71
10. *Defining aims and shared vision*	64	10. *Funding*	69

#### Comparison of prospective versus retrospective approaches

The top ten constructs for examining sustainability throughout an initiative (prospective assessment) versus after implementation (retrospective assessment) are presented in Table 5. Several key differences are observed. Prospective approaches are used for a combination of planning, guidance and evaluation. Prospective approaches show a greater emphasis on *building relationships* and getting *stakeholder buy-in* throughout an initiative. These approaches also highlighted the role of *initiative adaptation* to ensure initiatives align with stakeholder and setting needs. Retrospective approaches were more often designed for evaluation purposes emphasising the need for *a shared vision* and *accountability* to deliver the initiative. These approaches were more likely to specifically examine *funding* for the initiative and highlight the need to have a *defined aim* to show evidence for sustainability of an initiative once it has been ‘completed’. These differences highlight how retrospective approaches tend to focus on delivery and evidence for continuation of initiatives while prospective approaches focus on building an initiative into an organisation, getting people on board and garnering networks that may help along the way.

**Table 5:** Comparison of when to assess (differences shown in italics)

Retrospective assessment (9 approaches)	Percent	Prospective assessment (41 approaches)	Percent
1. Demonstrating effectiveness	100	1. Resources_general	93
2. Resources_general	89	2. Demonstrating effectiveness	85
3. Leadership and champions	89	3. Monitoring progress over time	83
4. *Accountability of roles and responsibilities*	78	4. *Stakeholder participation*	83
5. *Belief in the initiative*	67	5. Integration with existing programs and policies	81
6. *Defining aims and shared vision*	67	6. Training and capacity building	78
7. *Funding*	67	7. *Intervention adaptation and receptivity*	73
8. Monitoring progress over time	67	8. Leadership and champions	73
9. Training and capacity building	67	9. Belief in the initiative	68
10. Integration with existing programs and policies	67	10. *Relationships and collaboration and networks*	68

## Discussion

This review aimed to identify available approaches which assess or influence sustainability in healthcare and explore what sustainability constructs were examined in each to inform their future use in practice. This review found that a substantial number of approaches exist with 62 approaches identified and included in this review. Approach characteristics were wide-ranging with diverse settings, interventions and designs. Each provided a unique perspective on sustainability with no two being exactly alike.

The reviewed sustainability approaches made connections to many different theoretical perspectives which highlighted the complexity of measuring and planning for sustainable initiatives. Four theoretical perspectives (diffusion of innovations theory, complexity theory, ecological theories and open systems theory) were most common and revealed two distinct positions guiding the use of sustainability approaches. The first views sustainability as a linear process following implementation. In this approach, sustainability is an end goal, a state to be reached or level of achievement [70]. The second views sustainability concurrent process alongside implementation, where sustainability is a process to be influenced and adapted to impact initiative longevity [71-73]. Value is seen in both views, but depending on what theoretical perspective is taken, planning, measurement and monitoring is significantly different [8, 13, 61, 74, 75]. Despite previous work finding that ‘most frameworks proposed tend to be deterministic in nature where sustainability is viewed as an end goal’, we found that 66% of approaches we reviewed saw sustainability as a process rather than an end state [15]. The choice to evaluate, monitor or plan for sustainability overtime rather than after implementation may indicate a shift in perspectives from sustainability as an outcome to sustainability as an ongoing process. As this perspective gains popularity, some have cautioned that while it may be valuable to assess sustainability throughout initiatives, data collection past the implementation stage is still required to assess the continuation of initiative activities or outcomes and determine whether sustainability is actually achieved [18]. This highlights the need for the purpose of use to be clear before an approach is applied. While some approaches explicitly aim to sustain outcomes, others are meant to influence and promote action overtime. Therefore, the aims and potential results from approaches should be understood to ensure people are able to realistically assess the outcomes they desire.

Results have demonstrated that sustainability is most often defined and assessed as the *maintenance of programme activities*. Although multiple definitions were found (continuation of the health benefits from an initiative, capacity built in the workforce or community, further development or adaptation and the ability to recover costs*)*, there was a clear dependence on this one measure which has been previously observed in the literature [76]. It is important to note that while measuring continuation of programme activities is important to assessing sustainability, relying solely on this measure may risk other key sustainability variables being missed [18, 76]. For example, it may result in the continuation of ineffective or undesirable practices if health benefits are not taken into account. This was observed in the Drug Assistance Resistance Education programme in America which continued to be implemented in schools despite studies showing that it had little effect on prevention or reduction of drug use by students [77]. Using continuation of programme activities as the sole measure of sustainability also risks initiative being unfairly judged as failing to sustain if activities are adapted. If the definition is broadened, adaptation could also signify sustained improvement, especially if the adaptations contributed further to health benefits or cost recovery. These examples highlight the need for careful consideration of what will be sustained and what evidence there is for sustainability to occur [66]. All definitions identified in the review represent interrelated facets of what sustainability means in practice; therefore, those working in this field should explore the breadth of available sustainability domains in order to accurately represent the sustainability process and account for its full complexity and possible outcomes [7].

Our comparison across approaches demonstrated consistent constructs were seen regardless of proposed interventions, setting or level of application. Within the six constructs included in the majority of approaches, diverse views and different assessment mechanisms were taken, highlighting the complexity within each construct. This demonstrates the need for careful planning and consideration of how each construct is articulated and assessed given the specific outcomes of interest desired. Interestingly, no approaches contained the same combination of the constructs nor did any single method capture all identified constructs. Given homogeneity of the individual constructs found, we believe there is value in having an overarching resource and summary, indicating the breadth of possible sustainability constructs to consider for sustainability in healthcare settings. The consolidated framework for sustainability constructs in healthcare (Table 3) provides a knowledge base for those who may wish to review proposed sustainability constructs and draw on the substantial work and research already conducted in this area.

The framework can also help those considering creating a sustainability method in their own setting. While there are benefits of approaches created for specific settings, there is also a risk in continually creating ‘new’ approaches with similar constructs divided by semantics and personal interpretations of the literature [18, 20]. Those considering creating a sustainability approach should consider the information presented here and the available approaches for use before ‘recreating the wheel’ as continuous production may lead to further division and confusion in the literature and ultimately result in fewer robust studies on the use of available sustainability approaches being published [18]. The number of sustainability approaches may grow with necessary alterations to design and further development, but there is a need for future authors to describe how new approaches fit within the findings presented here. Authors should explicitly state how approaches have been created (particularly drawing on previous approaches which have informed the development) and highlight if they are transferable to other settings and if there are any specific benefits or barriers to their use.

### Strengths and limitations

This is the first review to consolidate available approaches for sustainability across diverse healthcare settings. We believe this work represents a significant contribution to the field in organising and describing sustainability approaches which have until now remained isolated across healthcare fields and disciplines [18]. This review provides not only a resource for identifying available sustainability approaches but also outlines the aims, applications and constructs in each approach so readers can determine if one may be fit for their setting. This work has demonstrated that although many approaches were developed within specific interventions and settings, similar constructs for sustainability were found indicating general learning can be gathered from across settings to inform sustainability processes and research. Additionally, this paper provides a consolidated summary of all constructs deemed to be important across approaches to serve as a sustainability knowledge base that is useful beyond specific settings or interventions. To aid readers in navigating the data extracted from each approach, we propose a list of questions to guide their decision-making process (Table 6). Readers can respond to these questions and use their responses along with full method details in Additional file 1 to establish if an available method will suit their purposes.

**Table 6:** Questions for consideration

Navigating available sustainability approaches—questions for consideration	
1. How do you wish to view sustainability? (a process or an end goal) 2. What is your aim? (evaluation, planning, guidance) 2. What does sustainability mean to you? (continuation of the health benefits, continuation of activities, capacity built, further development and/or cost recovery) 3. Where do you wish to use the sustainability approach? (specific intervention or organisation) 4. Who will use the approach? (researcher, practitioner, managers etc.) 5. Does an existing approach meet your needs? 6. If not, what needs to change or be adapted and why?	

The use of one author to conduct of the majority of screening, data extraction and coding is also a limitation of this work. Although double data extraction is recommended in most systematic reviews, it is also recognised that this is often not possible in many cases due to time and resources constraints [78]. This may have resulted in bias in inclusion or exclusion or resulted in missing or erroneous information being collected. To address this limitation, we involved multiple authors where possible in selection of the studies (20% screened by a second author) and coding of constructs (25% of studies). Data extraction was also checked against full-text articles for all included papers.

Another limitation of this work is the disproportionate number of frameworks from the community health and public health settings. These areas tend to dominate this area of research so further work may be needed to explore sustainability in other acute and chronic care settings [79].

Another key limitation of this work is that we did not use an existing quality assessment tool and cannot attribute value or accuracy of constructs from each approach. While the quality criteria set out in our data extraction form allowed us to ensure each paper had a minimum level of data to adequately describe the approach, it did not assess quality of the approaches themselves. We extracted information on each of the approaches which others may wish to use to attribute validity to findings. Details, particularly those around sustainability approach development, may be used by readers to assess whether they believe the approach has enough merit to be used in their site. It is important to note that many approaches (24%) were informed by professional expertise, a technique that may be difficult to assess for quality but appears to be very significant in the creation of sustainability approaches.

We reported which constructs were deemed to be important to assess, but this does not indicate that these are the ‘right’ constructs or that they will lead to sustainability. Although our assessment of frequency indicated some consensus across approaches, with six constructs included in over 75% of approaches, this does not tell us that assessing these constructs will achieve sustainability in practice or that they are correct or comprehensive. In order to understand the validity of these findings, the approaches must be applied and assessed in practice. Future work will explore if and how these approaches have been applied to ascertain if their constructs accurately represent sustainability in specific settings and if they fulfil their stated aims.

### Future work

Many approaches presented in this review recommend that they be used and evaluated further within other healthcare initiatives and settings to explore applicability and further development needed [11, 36, 56, 79]. Future work in this field should now focus on applying the available approaches in practice to understand the application processes and assess the overall impact of their use [18].

### Conclusion

Sustainability of improvements has been recognised as a challenge for some time, and while there is diversity in the literature on how it is defined and how it can be influenced, there is one clear and compelling message: sustainability of initiatives requires thoughtful planning and attention. If we do not address it appropriately, we continue to risk wasting valuable resources and losing significant progress and patient outcome improvements. Choosing a sustainability approach to support this process can pose a challenge to those looking to influence sustainability because of the diverse approaches reported in the literature. Understanding the purpose, perspectives and constructs within each will aid potential users to make the most of approach choice and application.

## Additional files

Additional file 1: Data extraction form. Full data extraction details for each method. (XLSX 30 kb)

Additional file 2: Definition and description of sustainability constructs. Table provides definitions, descriptions and examples for each of the 40 sustainability constructs found constructs. (PDF 446 kb)
