# Supplementary material for: Navigating the sustainability landscape: a systematic review of sustainability approaches in healthcare
Source: Implement Sci. 2018 Feb 9;13:27. doi: 10.1186/s13012-017-0707-4 (PMC5810192; doi:10.1186/s13012-017-0707-4)
Supplement: Supplementary file 2 — Definition and description of sustainability constructs. Table provides definitions, descriptions and examples for each of the 40 sustainability constructs found constructs. (PDF 446 kb) [file 13012_2017_707_MOESM2_ESM.pdf]

Additional File 2: Descriptions, Definitions and Examples of Sustainability Constructs

Theme	Construct	Description of Inclusion	Definition	Example
The External Environment	Awareness and Raising the profile	• Business cases<br>• Active Communication of main messages and benefits<br>• Publicity- marketing and sales pitch<br>• Newsletters, publications<br>• Success sells<br>• Awareness of the issue	Ensuring that stakeholder such as the community are aware of the initiative and its benefits and strategic steps are taken to raise the profile of the project to garner further support though media, marketing and publications.	*'Communication of value added (business case made)'[1]*
	Socioeconomic and political considerations	• Environmental considerations<br>• Government policies<br>• Social indicators<br>• Critical events<br>• Technology change<br>• Market forces<br>• Population characteristics<br>• Environmental scans	Awareness of the potential impact of outside forces in the environment, government or society that may impact initiative funding, processes or priorities	*'The program has political support from outside of the organization.'[2]*
	Spread to other organisations	• program diffusion<br>• replication at other sites<br>• spread to other parts of an organisation	Ability of an innovation to show benefits which are either spread within an organisation or to other organisations.	*'Another potential longer-term outcome from worthy innovative programs is that the underlying concepts or interventions them- selves may spread to other locations.'[3]*
	Urgency	• Scale of the burden<br>• urgency of the issues<br>• Severity of the issue<br>• relevance to fundamental mandates of the organisation<br>• concern for the problem	The urgency or motivation to maintain an initiative based on its potential to support an important and relevant healthcare need.	*'Urgency and the scale of the socio- economic burden'[4]*
Negotiating Initiative processes	Accountability of roles and responsibilities	• Tasks, activities, roles taken on, delegation,<br>• professional responsibilities<br>• varied roles for participation,<br>• allocation of work between individuals,<br>• burden to individuals<br>• proportion of staff contributing<br>• clarity of roles and responsibilities<br>• reliance on individuals	Roles and responsibilities involved in the initiative are clearly defined and outlines with necessary distribution across teams as necessary so there is no reliance on specific individuals.	*'Roles and responsibilities of service staff to initiate change established'[5]*
	Belief in the initiative	• Value of the initiative<br>• legitimacy of the intervention,<br>• confidence in the	The belief that, the initiative will be of value, it will produce the benefits intended	*'Do staff think that the change is a better way of doing things that they want to preserve for the future?'[6]*

		intervention<br>• commitment of stakeholders<br>• staff perceptions of need and quality of innovation<br>• credibility, utility	and deliver the stated improvements to care.	
	Complexity	• Burden (complexity and cost)<br>• Program familiarity<br>• simplicity, easy to describe and teach	The difficulty and complexity of understanding, delivering and maintaining the initiative.	*'Despite the usual exceptions in practice it is not hard to perform the work practice as prescribed.'[7]*
	Defining Aims and Shared Vision	• Consensus, shared goals<br>• clarifying goals, set directions, shared purpose,<br>• mutual goal setting,<br>• shared expectations	Taking the time to define and understand what people want to achieve and why. Working with stakeholders to establish a shared aim and vision.	*'They spoke of the need for the goals to be developed by both the visitors and the host country partners as well as goal revision during the actual exchange experience.' [8]*
	Incentives	• Positive incentives to participate<br>• individual benefits,<br>• perceived effects,<br>• higher prestige, increased reputation for an institution, awards, and process performance feedback<br>• rewards<br>• motivation	Motivation gained from rewards or benefits that drive individuals and organisations to engage with an initiative and continue to deliver it overtime.	*'Extrinsic benefits can be as obvious as the survival of the health care organization, keeping our job, in- creased recognition, receiving a promotion or a raise, increased interaction and learning from others, and improvements in patient care'[9]*
	Job requirements	• Work agreement,<br>• delegating responsibilities,<br>• job descriptions, job roles,<br>• skill sets,<br>• activities incorporated into day jobs	Specific job requirements have been established and included in job descriptions and roles which are able to be accomplished with the given skills set of workers.	*'Can implementation and sustainability tasks be part of key staff job descriptions?'[10]*
	Workload	• Sufficient number of staff,<br>• time to complete tasks in daily schedule,<br>• workload is manageable,<br>• added effort,<br>• reduce waste,<br>• division of labour	The added effort and change to workload when a new initiative in implemented. Staff have the necessary time to complete tasks and the initiative has fair divisions of labour and does not require special or extra effort.	*'The workload involved in using the ehealth system is manageable'[11]*
Resources	Resources_ General	• All resources, generic/unspecified mention of resources	Any resources needed to manage and maintain an initiative.	*'Resources invested in programmes'[12]*
	Funding	• Money<br>• Funding<br>• Grants<br>• Donations	Having adequate funding for the initiative to be implemented,	*'Strategic funding includes having plans and resources in place to support current and prospective program*

		• capital resources<br>• maximising billing,<br>• reimbursement	embedded and sustained.	*requirements.'[13]*
	Infrastructure	• Infrastructure support<br>• Workplace climate and structure,<br>• work spaces,<br>• materials, supplies and medications<br>• information support systems, software,<br>• access to information	The resources required to support the initiative to be delivered such as buildings, office space, materials and supplies.	*'Provide resource supports such as office space and staffing support.'[14]*
	Resource_Staff	• Sufficient number of staff<br>• Permanent personnel<br>• Human resources,<br>• staff mobility<br>• turnover	Having a sufficient number of staff to meet the requirements of the initiative.	*'Are the internal/external human resources in place to sustain the effort going forward (describe—e.g., team, leadership/champion)?' [15]*
	Resource_Time	• Time in schedules,<br>• staff time,<br>• volunteer time,<br>• allocated time	Energy and time to dedicate to the initiative	*'Time in daily schedule.' [16]*
The Initiative Design and Delivery	Demonstrating effectiveness	• Value<br>• Outcomes<br>• Success<br>• Effectiveness<br>• Benefits<br>• Assessment<br>• Learning and feedback<br>• Results	Assessing or measuring project outcomes and impact	*'Patient-centered outcomes that the intervention should generate as a result of its use'[17]*
	Evidence base for the initiative	• Data available<br>• Performance<br>• Strength of the evidence base<br>• Evaluation reports<br>• Credibility<br>• Research evidence	The evidence that the initiative will provide stated benefits and can credible achieve them through the initiative plan.	*'The program provides strong evidence to the public that the program works.'[2]*
	Expertise	• Special skills or experience,<br>• Clinical, technical, quality improvement,<br>• Creating and transferring expertise	Having adequate expert knowledge and experience to carry out an initiative.	*'External advisory boards create a different oversight by providing expertise'[18]*
	Improvement Methods	• PDSAs<br>• Continuous quality improvement<br>• Improvement processes	The use of improvement methods to support initiative success and sustainability	*'Using improvement methodology and measurement of its outcomes' [19]*
	Monitoring progress over time	• Audit and feedback<br>• Regular reporting mechanisms<br>• Reliable and relevant measures<br>• Progress and changes overtime<br>• Performance data	The ability to monitor the initiative using standardised systems or mechanisms over-time	*'Appropriate steps to gather and report data for health needs and programme eff effectiveness.'[20]*
	Project duration	• Timeframe<br>• Funding period<br>• Number of years support	How long the project is expected to last and how long	*'Time frame for sustainability assessment'[21]*

		is provided	resources are available.	
	Project type	• Project design<br>• Implementation plans<br>• Type-curative/preventative	The type and design of the initiative	*'What type of project is it (e.g. preventive versus curative)?'[22]*
	The Problem	• Disease burden<br>• social narrative around the problem<br>• Severity	The recognition, concern and acceptance of a problem that supports an initiative to address it.	*'Awareness of a problem, concern for a problem.'[23]*
	Training and Capacity Building	• Staff capacity<br>• Skills to deliver initiative<br>• Employee development<br>• Confidence to deliver<br>• Education sessions<br>• Peer training<br>• Level and experience of staff	Orienting and Training staff to be able to deliver the initiative successfully, as well as putting ongoing educational and skill building support in place for new workers.	*'Offer training to new staff and ongoing training to all staff including face-to-face meetings to promote staff mentoring opportunities'[14]*
The Organisational Setting	Integration with existing programs and policies	• Ability to fit in with organisations,<br>• how the intervention 'conforms' to existing institutions,<br>• integration into routine processes,<br>• alignment with strategic goals, administrative policies and procedures	The need to ensure an initiative was embedded into organisational structures, programmes and policies.	*'Integration within existing or realizable patterns of service organization and delivery.'[24]*
	Intervention Adaptation and receptivity	• Response to opportunities and challenges,<br>• modification,<br>• flexibility,<br>• monitored and evaluated regularly, adjusted as needed	The ability of an initiative to respond to change and adapt to fit with local contexts and requirements.	*' The reciprocal fit of an intervention within a practice setting and the practice setting within the larger ecological system.'[17]*
	Opposition	• Competing priorities,<br>• opposition from stakeholders	Resistance from stakeholders to the initiative due to other priorities or competing interests.	*'The presence (or absence) of organized opposition.'[25]*
	Organisational Readiness and Capacity	• Viability,<br>• Abilities and experience, preparedness,<br>• building capacity/education,<br>• Absorptive capacity,<br>• support service provision,<br>• Ability to learn and adapt to the environment	Capacity and readiness of the organisation to undertake the initiative.	*'Readiness of health facilities and their outreach workers to provide services'[26]*
	Organisational Values and Culture	• Prevailing beliefs and value systems,<br>• compatibility of beliefs with initiative,<br>• support for	Organisational beliefs and values that support a culture for the initiative and its sustainability.	*'Strategic execution: demonstrating highly visible and maintained support of the change/ transformation, communicating progress to all stakeholders, and considering*

		improvement,<br>• limitations and opportunities created by the organisation,<br>• workplace climate and cohesion		*the adoption of the change/transformation as a top organization priority'[27]*
	Support Available	• Reminders,<br>• education boosters,<br>• management support,<br>• supervision and peer-support,<br>• technical assistance<br>• data support	Support in the form of reminders, staff, technical and educations, available to enhance delivery and maintenance of the initiative.	*'What means exist for informing programme drivers of changes in the health concern, its determinants, barriers to achievement of anticipated effects, or shortfalls in realising anticipated effects and engaging them in supporting change?'[20]*
The People Involved	Leadership and Champions	• Leadership depth, distribution, and continuity<br>• Leadership support and engagement<br>• Style<br>• Commitment to and Advocates for the initiative<br>• Experienced and strong leadership	A person, or group of people who have the ability and skills to can advocate, communicate and support an initiative to achieve lasting change.	*'Leadership concerns the willingness and ability of actors to commit to efforts; have a clear and long-term vision, gain political and financial support, build a programme's credibility, build trust and engage with various stakeholders, mobilize resources and implementation efforts, taking ownership of reforms and innovations.'[28]*
	Ownership	• Sense of ownership<br>• Community/Local ownership<br>• Local control and management	Organisations, communities and stakeholders taking ownership and responsibility to support, embed and sustain an initiative.	*'Establish and/or maintain sufficient ownership among innovation stakeholders'[29]*
	Power	• Power distribution<br>• Professional power<br>• Stakeholder power and priorities<br>• Power exchange	The ability of individuals to use their power to advocate or support the initiative.	*'Assess those who have power and act as advocates for the functional area related to the innovation'[29]*
	Relationships and collaboration and networks	• Community links<br>• Networking<br>• Building alliances<br>• Collaboration with other scientists, organisations, communities etc.<br>• Partnerships	Ability to build collaborations, partnerships and networks to support sustainability of the initiative	*'Part of the strategic planning process ought to be to create support networks among stakeholders.'[30]*
	Satisfaction	• Meaningful work,<br>• role satisfaction<br>• benefits of learning new tasks<br>• increase task significance<br>• job more intrinsically rewarding and person-ally significant<br>• Enjoyment and fun	The level of enjoyment and reward stakeholders and staff get from participating in the initiative.	*'Factors that promote or inhibit actors' apprehension of a practice as meaningful.'[31]*
	Stakeholder participation	• Involvement of those impacted by the initiative,<br>• Engagement<br>• Valuing stakeholder opinions	The need for involvement and participation from stakeholders who are affected by the initiative	*'How do key stakeholders influence the programme and what guides their decisions?'[20]*

		• Influencing the initiative		
	Community participation	Broad based participation by the community<br>Community dialogue<br>Community voice<br>Understanding the community, local context, local needs and values<br>Cultural acceptance<br>Interest and support	Participation of community members to direct, and shape initiatives to reflect their values, expectations and needs.	*'Are project approaches and goals discussed with recipient community members, as equal partners?'[22]*
	Patient involvement	• Patient centeredness<br>• Patient choice and preferences<br>• Beneficiary perspectives considered<br>• Patient values<br>• Empowerment	Involving patients in initiative processes to understand potential impact, values and preferences.	*'Are there appropriate steps to include a beneficiary perspective?'[20]*
	Staff involvement	• Building staff capacity to deliver the initiative<br>• Involvement in decision making, planning and development of initiatives<br>• Staff interest<br>• Proportion of staff that contribute<br>• Staff attitudes and acceptance	Including staff responsible for implementing an initiative across multiple stages of planning, design, delivery and maintenance, valuing their input and taking feedback on board.	*'Staff involvement and integration is the inclusion of committed, qualified staff in program design, implementation, evaluation, and decision making.'[13]*

References:

1. Alexander JA, Weiner BJ, Metzger ME, Shortell SM, Bazzoli GJ, Hasnain-Wynia R, et al. Sustainability of collaborative capacity in community health partnerships. Med. Care Res. Rev. 2003;60:130S–160S.

2. Luke D a, Calhoun A, Robichaux CB, Elliott MB, Moreland-Russell S. The Program Sustainability Assessment Tool: a new instrument for public health programs. Prev. Chronic Dis. 2014;11:130184.

3. Scheirer MA, Dearing JW. An agenda for research on the sustainability of public health programs. Am. J. Public Health. 2011;101:2059–67.

4. Atun R, de Jongh T, Secci F, Ohiri K, Adeyi O. Integration of targeted health interventions into health systems: a conceptual framework for analysis. Heal. Policy Plan. (C) Copyright Oxford University Press 2010.: (1)Professor of International Health Management, Imperial College, London, UK, (2)Researcher, Centre for Health Management, Imperial College London, UK, (3)Doctoral Researcher, Centre for Health Management, Imperial College London, UK, (4)Health Specialis; 2010. p. 104–11.

5. Blackford J, Street A, J. B. Tracking the route to sustainability: A service evaluation tool for an advance care planning model developed for community palliative care services. J. Clin. Nurs. Copyright (C) 2012 Blackwell Publishing Ltd.: Blackwell Publishing Ltd (9600 Garsington Road, Oxford OX4 2XG, United Kingdom); 2012. p. 2136–48.

6. Maher L, Gustafson D, Evans A. Sustainability Model and guide. Coventry, UK: NHS Institute for Innovation and Improvement; 2010.

7. Slaghuis SS, Strating MMH, Bal R a, Nieboer AP. A framework and a measurement instrument for

sustainability of work practices in long-term care. BMC Health Serv. Res. BioMed Central Ltd; 2011;11:314.

8. Leffers J, Mitchell E. Conceptual Model for Partnership and Sustainability in Global Health. Public Heal. Nurs. Copyright (C) 2011 Blackwell Publishing Ltd.: Blackwell Publishing Inc.; 2011. p. 91–102.

9. Persaud D. Enhancing Learning, Innovation, Adaptation, and Sustainability in Health Care Organizations: The ELIAS Performance Management Framework. Heal. Care Manag. (C) 2014 Wolters Kluwer Health | Lippincott Williams & Wilkins: Author Affiliation: School of Health Administration, Dalhousie University, Halifax, Canada.; 2014. p. 183–204.

10. Feldstein AC, Glasgow RERE. A Practical , Robust Implementation for Integrating Research Findings into Practice. Jt. Comm. J. Qual. patient Saf. 2008;34:228–43.

11. Finch TL, Mair FS, O'Donnell C, Murray E, May CR, T.L. F, et al. From theory to "measurement" in complex interventions: methodological lessons from the development of an e-health normalisation instrument. BMC Med. Res. Methodol. England; 2012;12:2–16.

12. Amaya AB, Caceres CF, Spicer N, Balabanova D, Amaya AB, Caceres CF, et al. After the Global Fund : Who can sustain the HIV / AIDS response in Peru and how ? Glob. Public Health. Taylor & Francis; 2014;9:176–97.

13. Mancini JA, Marek LI. Sustaining Community-Based Programs for Families: Conceptualization and Measurement. Fam. Relat. 2004;53:339–47.

14. Ford JH, Alagoz E, Dinauer S, Johnson KA, Pe-Romashko K, Gustafson DH. Successful Organizational Strategies to Sustain Use of A-CHESS: A Mobile Intervention for Individuals With Alcohol Use Disorders. J. Med. Internet Res. 2015;17:e201.

15. Edwards JC, Feldman PH, Sangl J, Polakoff D, Stern G, Casey D. Sustainability of partnership projects: A conceptual framework and checklist. Jt. Comm. J. Qual. Patient Saf. 2007;33:37–47.

16. Dominick GM, Tudose A, Pohlig RT, Saunders RP. Sustainability of physical activity promoting environments and influences on sustainability following a structural intervention in residential children ' s homes. 2016;31:207–19.

17. Chambers D a, Glasgow RE, Stange KC. The dynamic sustainability framework: addressing the paradox of sustainment amid ongoing change. Implement. Sci. Implementation Science; 2013;8:117.

18. Dorsey SG, Schiffman R, Redeker NS, Heitkemper M, McCloskey DJ, Weglicki LS, et al. National Institute of Nursing Research Centers of Excellence: a logic model for sustainability, leveraging resources, and collaboration to accelerate cross-disciplinary science. Nurs. Outlook. United States; 2014;62:384–93.

19. Parand A, Benn J, Burnett S, Pinto A, Vincent C. Strategies for sustaining a quality improvement collaborative and its patient safety gains. Int J Qual Heal. Care. (C) International Society for Quality in Health Care and Oxford University Press 2012. Published by Oxford University Press. All rights reserved.: Department of Surgery and Cancer, Imperial College London, Rm 503, 5th Floor Wright Fleming Building, St Mary's Campus, Norfolk Place, London W2 1PG, UK; 2012. p. 380–90.

20. Gruen RL, Elliott JH, Nolan ML, Lawton PD, Parkhill A, McLaren CJ, et al. Sustainability science: an integrated approach for health-programme planning. Lancet. Elsevier Ltd; 2008;372:1579–89.

21. Saunders RP, Pate RR, Dowda M, Ward DS, Epping JN, Dishman RK. Assessing sustainability of Lifestyle Education for Activity Program (LEAP). Health Educ. Res. 2012;27:319–30.

22. Shediac-Rizkallah MC, Bone LR. Planning for the sustainability of community-based health programs: conceptual frameworks and future directions for research, practice and policy. Health Educ. Res. 1998;13:87–108.

23. Goodman RM, Steckler A. A model for the institutionalisation of a health promotion program.pdf. Fam Community Heal. 1989. p. 63–78.

24. May C. A rational model for assessing and evaluating complex interventions in health care. Bio Med Cent. Heal. Serv. Res. 2006;6:11.

25. Nelson DE, Reynolds JH, Luke D a, Mueller NB, Eischen MH, Jordan J, et al. Successfully maintaining program funding during trying times: lessons from tobacco control programs in five states. J. Public Health Manag. Pract. 2007;13:612–20.

26. Sarriot E, Yurkavitch J, Ryan L, The Sustained Health, Outcomes (SHOUT) Group. Taking the Long View : A Practical Guide to Sustainability Planning and Measurement in Community- Oriented Health Programming. Manual. Calverton, MD.; 2008.

27. Schalock RL, Verdugo M, Lee T. A systematic approach to an organization's sustainability. Eval. Program Plann. 2016;56:56–63.

28. Shigayeva A, Coker RJ. Communicable disease control programmes and health systems: An analytical approach to sustainability. Health Policy Plan. 2015;30:368–85.

29. Johnson K, Hays C, Center H, Daley C. Building capacity and sustainable prevention innovations: a sustainability planning model. Eval. Program Plann. 2004;27:135–49.

30. Brinkerhoff DW, Goldsmith AA. Promoting the sustainability of development institutions: A framework for strategy. World Dev. 1992;20:369–83.

31. May C, Finch T. Implementing, Embedding, and Integrating Practices: An Outline of Normalization Process Theory. Sociology. 2009;43:535–54.
